# Comparative organizational research starts with sound measurement: Validity and invariance of Turker’s corporate social responsibility scale in five cross-cultural samples

**DOI:** 10.1371/journal.pone.0207331

**Published:** 2018-11-19

**Authors:** Daniel Schulze, Kathrin Heinitz, Timo Lorenz

**Affiliations:** 1 Department of Education and Psychology, Freie Universität Berlin, Berlin, Germany; 2 Department of Psychology, Medical School Berlin, Berlin, Germany; Universita degli Studi di Pisa, ITALY

## Abstract

As organizational research turned its focus to Corporate Social Responsibility (CSR), interest also grew in the individual’s perspective on CSR. When looking for cross-cultural comparisons of the effects of CSR, measurement invariance is of utter importance as a questionnaire might not be equivalent in all investigated samples and thus bias results. We examined a previously published questionnaire assessing different aspects of personal CSR ratings. Factorial validity and measurement invariance was tested by means of confirmatory factor analysis and Bayesian structural equation modeling in five samples (total N = 1120): 2 US-American, 2 German, and 1 English-speaking Indian sample. In an exploratory-confirmatory approach, the originally proposed factor structure was altered to finally comprise four facets of CSR: employee-related CSR, environmental CSR, philanthropy and customer-related CSR. Measurement invariance tests showed evidence for small differences of the English and German version as well as significant divergences of the measurement model in the Indian sample. In conclusion, we show the validity of the questionnaire for a circumscribed Western context but are hesitant about further transfers. Future research on perception of CSR in non-western contexts might depend on new and tailored questionnaires.

## Introduction

Interest in Corporate Social Responsibility (CSR) is widely growing [1,2]. Organizations engage in CSR due to various reasons, such as stakeholder pressure [3,4], expectations of improved reputation [5], competitive advantage [6] or higher business returns [7,8] as well as a sense of responsibility [9]. CSR refers to “context-specific organizational actions and policies that take into account stakeholders’ expectations and the triple bottom line of economic, social, and environmental performance” [10]. Although it is mostly examined on an institutional or organizational level [11], CSR is also an important factor on the individual level. Employees can be considered as key internal stakeholders as it is them who have to implement CSR into daily business [12,13]. Therefore their individual perception of CSR can be crucial to CSR success. Research on this employee-focused micro-CSR is on the rise [14,15] and as this research often implies the employee’s perspective on the organizations’ CSR initiatives, instruments are in need that reliably and validly measure this perspective [16].

Several instruments are currently applied to measure different facets of employee perceptions of CSR or related constructs such as corporate citizenship [2,17,18]. Turker [19] proposed a measure of CSR that is based on the definition of CSR as "corporate behaviors that aims to affect stakeholders positively and that go beyond its economic interest" [19] and displays four facets: "CSR to social and nonsocial stakeholders, employees, customers, and government" [19]. Her general scale development was based on the stakeholder typology of Wheeler and Sillanpää [20], the item development was based on previous scales and additionally included newly developed items [19]. Turker’s measure differentiates various aspects of CSR in contrast to shorter questionnaires with a global approach to CSR, e.g., [15]. The measure has since been used in several studies, e.g., [21–26], however, the factorial structure has barely been validated, although Turker herself noted that “there is a need for further studies to confirm the current structure of the scale” [19]. The aim of this paper therefore is to scrutinize the factorial validity of a CSR measure on the individual level.

## Method

The original scale was developed in Turkey, a country according to Turker having “a unique position between Eastern and Western countries” [19]. We therefore assessed persons with different cultural backgrounds in order to examine if the factorial structure holds in differing cultural contexts.

### Participants and procedure

The first US sample (US-1) had a total of 146 participants (77 women, 69 men) between 20–62 years of age (M_age_ = 36.89 years, SD_age_ = 11.40, Mdn_age_ = 33 years), which were recruited via a survey link in social networks (32 respondents) and by using Amazon mechanical Turk (114 respondents; for the usability of Amazon mechanical Turk see e.g., [27,28]). The participants had higher education on average with 65% holding a bachelor’s degree or higher. IT and media was the most common corporate sector with 17%, 67% had a permanent contract. The second US sample (US-2) had a total of 194 participants (74 women, 120 men) between ages 20–59 (M_age_ = 34.48 years, SD_age_ = 10.06, Mdn_age_ = 31.5 years), which were recruited using Amazon mechanical Turk. Again, most participants had higher education (63% obtained a bachelor’s degree or higher), worked in IT and media (15%) and had a permanent contract (80%). Both US surveys were administered in English.

The first German sample (GER-1) comprised 155 participants (96 women, 59 men) between ages 20–59 (M_age_ = 33.63 years, SD_age_ = 8.62, Mdn_age_ = 33 years). A bachelor’s degree or higher was achieved by 58%. The service sector was the most frequent (21%) and 62% had a permanent contract. The second German sample (GER-2) consisted of 193 participants (123 women, 70 men), age categories 18–24 to 60+ (Mdn_age_ = 25–29 years). Here, a bachelor’s degree or higher was achieved by 52%. The social and health sector was the most frequent (24%), followed by the service sector (20%), and 65% had a permanent contract. Participants in both samples were recruited by spreading the survey link via social networks, e.g. Facebook or Xing. The surveys were administered in German.

The fifth sample was collected in India (INDIA) and had a total of 432 participants (148 women, 284 men) between ages 17–71 (M_age_ = 30.5 years, SD = 6.23, Mdn_age_ = 29). The data used in this study is derived from an employee attitude survey in India, particularly in Bangalore, Delhi and Mumbai and by e-mails sent out to members of India’s largest network on Corporate Sustainability and Corporate Social Responsibility. Thus, the sample comprised highly educated employees (92% had a bachelor’s degree or higher) and 30% were in a supervising position within their company. Corporate branches were diverse with IT and media (28%) being the most frequent. The survey was administered in English.

Participation in all studies was strictly voluntary; no compensation was supplied but the Amazon mechanical Turk users who received 50 cents (sample US-1) or 1 dollar (sample US-2) for their participation in the survey. Participants were informed in written form that their data was obtained and analyzed anonymously and that they could stop the survey at any time.

This study is in accordance with the APA ethical principles regarding research with human participants. The study does not involve any conflict of ethics, since no clinical intervention was performed. Neither were blood or tissue samples taken for study purposes. Participants were informed before participating that their responses would be treated confidentially and anonymously and that all data would be analyzed in a generalized manner so that no conclusions could be drawn about individual persons. The participants were informed that they would give their consent by proceeding past the welcome page of the online survey. This procedure is in accordance with the Freie Universität Berlin ethics committee’ s guidelines. There was no contact between researchers and participants. Participation in this study was voluntary. The study was approved by the ethics committee of the Freie Universität Berlin ID 135/2017.

### Materials

Beside the demographics gender, age, education, and occupation, the items introduced by Turker [19,25] were presented, although marginally altered (e.g., "company" replaced by "organization") to suit a wider spectrum. The samples used were all part of several yet unpublished studies concerning the individual level effects of CSR. Therefore further scales were part of the respective surveys. Topics of the additional scales comprised affective commitment, organizational cynicism, organizational citizenship behavior, work deviance behavior, meaning of work, organizational identity, engagement or pro-environmental behavior.

The German translation of the scale was close to the original, as it was developed using a standard translation-back-translation procedure [29]. Table 1 shows both versions of the scale. It is important to note, however, that we did not use the two items concerning CSR to government, as these items do not reflect self-motivated CSR actions but mere adherence to laws and regulations [30]. Thus, the questionnaire presented to the participants comprised 15 items with a five point Likert scale from 1 = strongly disagree to 5 = strongly agree. As all participants had to fill in all items in order to finish the questionnaire, no missing data was present.

**Table 1:** Items wordings used in this study.

Item	English wording	German wording
1	My organization participates in activities which aim to protect and improve the quality of the environment.	Mein Unternehmen unterstützt Aktivitäten zur Verbesserung und zum Schutz der Natur.
2	My organization invests in order to create a better life for future generations.	Mein Unternehmen investiert, um ein besseres Leben für zukünftige Generationen zu sichern.
3	My organization implements special programs to minimize its negative impact on the environment.	Mein Unternehmen führt Maßnahmen durch, um den negativen Einfluss auf die Umwelt zu minimieren.
4	My organization targets sustainable growth which takes future generations into consideration.	Mein Unternehmen setzt auf nachhaltiges Wachstum, welches zukünftige Generationen berücksichtigt.
5	My organization supports non-governmental organizations working in problem areas.	Mein Unternehmen unterstützt Nicht-Regierungs-Organisationen, welche in Problemgebieten aktiv sind.
6	My organization contributes to campaigns and projects that promote the well-being of society.	Mein Unternehmen trägt seinen Teil zu Kampagnen und Projekten bei, die eine positive gesellschaftliche Entwicklung fördern.
7	My organization encourages its employees to participate in voluntary activities.	Mein Unternehmen ermutigt seine Mitarbeiter sich ehrenamtlich zu engagieren.
8	My organization’s policies encourage employees to develop their own skills and careers.	Mein Unternehmen fördert die Fähigkeiten und Karrieren seiner Mitarbeiter.
9	The management of my organization is primarily concerned with employees’ needs and wants.	Die Führungsebene meines Unternehmens kümmert sich um die Bedürfnisse seiner Mitarbeiter.
10	My organization implements flexible policies that provide a good work and life balance for its employees.	Mein Unternehmen setzt flexible Arbeitszeitmodelle um, damit eine gute Balance zwischen Arbeit und Freizeit für die Mitarbeiter entsteht.
11	The managerial decisions related to the employees are usually fair.	Die Entscheidungen der Führungspersonen in Bezug auf Mitarbeiter sind meistens fair.
12	My organization supports employees who want to acquire additional education.	Mein Unternehmen unterstützt Arbeitnehmer, die sich weiterbilden möchten.
13	My organization protects consumer rights beyond the legal requirements.	Mein Unternehmen schützt Verbraucherrechte über die rechtlichen Anforderungen hinaus gehend.
14	My organization provides comprehensive and accurate information about its products to its customers.	Mein Unternehmen teilt den Kunden vollständige und richtige Informationen über seine Produkte mit.
15	Customer satisfaction is very important for my organization.	Kundenzufriedenheit ist für mein Unternehmen sehr wichtig.

### Statistical analysis

#### Factor analysis

As factorial validity was the main objective of this study confirmatory factor analysis (CFA) was used to evaluate the measurement model of Turker’s CSR scale [19]. Fit of the measurement models was tested using the criteria proposed by Hu and Bentler [31]. Augmenting the often oversensitive chi-square test these recommendations involve a standardized root-mean-square residual (SRMR) ≤ 0.08 in combination with at least one of the following fit indices: a root-mean-square error of approximation (RMSEA) ≤ 0.06, a lower bound of the 90% confidence interval of the RMSEA ≤ 0.06, or a comparative fit index (CFI) ≥ 0.95. The Satorra-Bentler adjusted chi-square was calculated to adjust for nonnormality [32]. This ML variant is reliable as long as more than three ordinal answer categories are displayed [33]. These analyses were conducted in R [34] using the package "lavaan" [35].

Additionally, Bayesian structural equation modeling (BSEM) was used as an alternative to traditional maximum-likelihood parameter estimation (for application examples see [36,37]). In multidimensional models, ordinary CFA assumes completely independent clusters of items. Item cross-loadings are thus regarded as fixed effects constrained to zero [38]. This assumption often is too strict for practical purposes, where small deviations from complete independence have no substantial theoretical impact [39] and a random effect model is favorable over fixed effects. In BSEM [40], the zero-fixed cross-loadings are treated as random effects with mean zero and a small variance, which allows for sample-wise minor divergences from independence. As this approach inflates the number of parameters to be estimated, ordinary maximum-likelihood estimation is not feasible. BSEMs thus utilize a Markov chain Monte Carlo (MCMC) algorithm that uses prior information about the parameters to determine the most likely values of loadings, means, and variances. Mathematically, a prior is the distribution of the desired parameter incorporating the researcher’s knowledge about that parameter where the distribution’s mean reflects its a priori most likely value and the distribution’s variance and shape reflects the certainty of that knowledge. MCMC algorithms will converge even for otherwise too complex structural equation models as long as the priors are informative enough.

Following from the MCMC estimation, a chi-square statistic is determined to evaluate the model fit with a credibility interval generated by the distribution parameters of the MCMCs. A credibility interval can be interpreted straight forward as the range of probable values of the estimated parameter [41]. The posterior predictive p-value (PPp) corresponds to the credibility interval as a test of model fit. Muthén and Asparouhov [40] propose the traditional Neyman-Pearson threshold of PPp < .05 being a valid indicator of model misfit, although Bayesian modeling typically relies on model comparisons instead of fixed cut-offs. Furthermore, information criteria like the DIC are employed for model comparison, with smaller DICs suggesting superior model fit [42].

It follows from the above that the size of the prior variance is of crucial importance in two ways: First, a large variance will more likely lead to non-convergence of the MCMC algorithm. Second, the explanatory power of a multidimensional model decreases as the prior variances of the cross-loadings increase. Initially, all models were tested with a cross-loading prior variance of .01 of a normal distribution enclosing mean zero. We then extensively utilized sensitivity analyses to investigate the impact of different prior variances on the model fit. The main purpose was to identify the "tipping point" of the prior variances, which was defined by variances leading to a PPp > .05. Finding the smallest prior variance yielding good model fit was important since smaller variances lead to stricter and explanatory stronger models that will be more easily discarded by the chi-square test [40]. The maximum variance used in the sensitivity analyses was 1, displaying an uninformative prior.

We calculated BSEMs using the routine implemented in MPlus, version 6.12 [43], which uses a Gibbs sampler for MCMC estimation (for further details see [40]). To assure model identification, latent variances were fixed to 1. The starting values of two MCMC chains were altered to ensure meaningfully converged estimations (seed for the reported results was 429). We determined convergence of multiple Markov chains using the Potential Scale Reduction statistic (PSR [44]), where a value below 1.1 is taken as indicator for estimation convergence. A maximum of 200,000 iterations was tested, models without sufficiently low PSR were considered as not converged.

#### Analysis of measurement invariance

CFA allows for tests of measurement invariance across groups by equalizing certain parameters of the model. Usually, several stages of invariance tests are applied. Initially, weak measurement invariance implies a similar factor structure (configural invariance) and equal factor loadings in all groups. These conditions are viewed as requirements for comparisons of regression slopes between groups [45] and are therefore sufficient in correlational studies. Augmenting weak invariance, the second stage of strong invariance refers to equal item intercepts whose differences reflect possible response biases. As a third step, strict invariance extends the latter and represents the equality of the item residuals. Some authors ask for the latter if latent mean scores are to be compared between groups [46], whereas others find strong invariance satisfactory [47]. To evaluate invariance, the sketched series of models was calculated and models were compared to a baseline model, which is the respective previous model [45]. In these comparisons, the change in the chi-square statistic was tested for statistical significance and the change in various fit indices was evaluated. Following Chen’s [45] recommendations for unequal sample sizes, we retained the hypothesis of loading invariance when the chi-square change was insignificant (α > .05), or a decrease in CFI < .010 was accompanied by an increase in RMSEA < .010 or an increase in SRMR < .030. When concerning intercept and residual invariance the same rules applied except an increase in SRMR < .005 [45].

BSEM was considered here again, as minor differences between the same item loadings or item means in two groups can be due to true variation of the parameter without any impact on the model’s meaning [40]. Thus, differences between groups can again be treated as random effects with mean zero and a small variance instead of fixed constants of zero. Given the five samples, ten pair-wise differences had to be estimated for every of the 33 parameters in the measurement model. Given this complexity, MCMC estimations did not converge given the criterion of PSR < 1.1 [44]. Pair-wise comparisons majorly did not converge as well implying the need for larger samples. Measurement invariance was thus only evaluated using maximum-likelihood CFA.

#### Reliabilities

We report several reliability coefficients. As Cronbach’s α is only appropriate for unidimensional questionnaires consisting of tau-equivalent items [48], α is clearly not a sufficient estimate of reliability in the case of multidimensional scales with correlated factors. When all items of the questionnaire are averaged to get a rough estimate of general CSR, as usually done in practice, McDonald’s ω_h_ [49] is a better choice [50]. This coefficient captures the systematic variance of the total score, when a given number of sub-facets is first partialed out. Cronbach’s α on the other hand uses all systematic variance from the g-factor as well as specific sub-factors. Beside this estimate of general reliability of the whole scale computed through a Schmid-Leiman-transformation [51], we provide coefficients for the single factors too, again in terms of McDonald’s ω which in this case is derived from the estimated factor loadings.

## Results

### Factor structure

**Fig 1:**
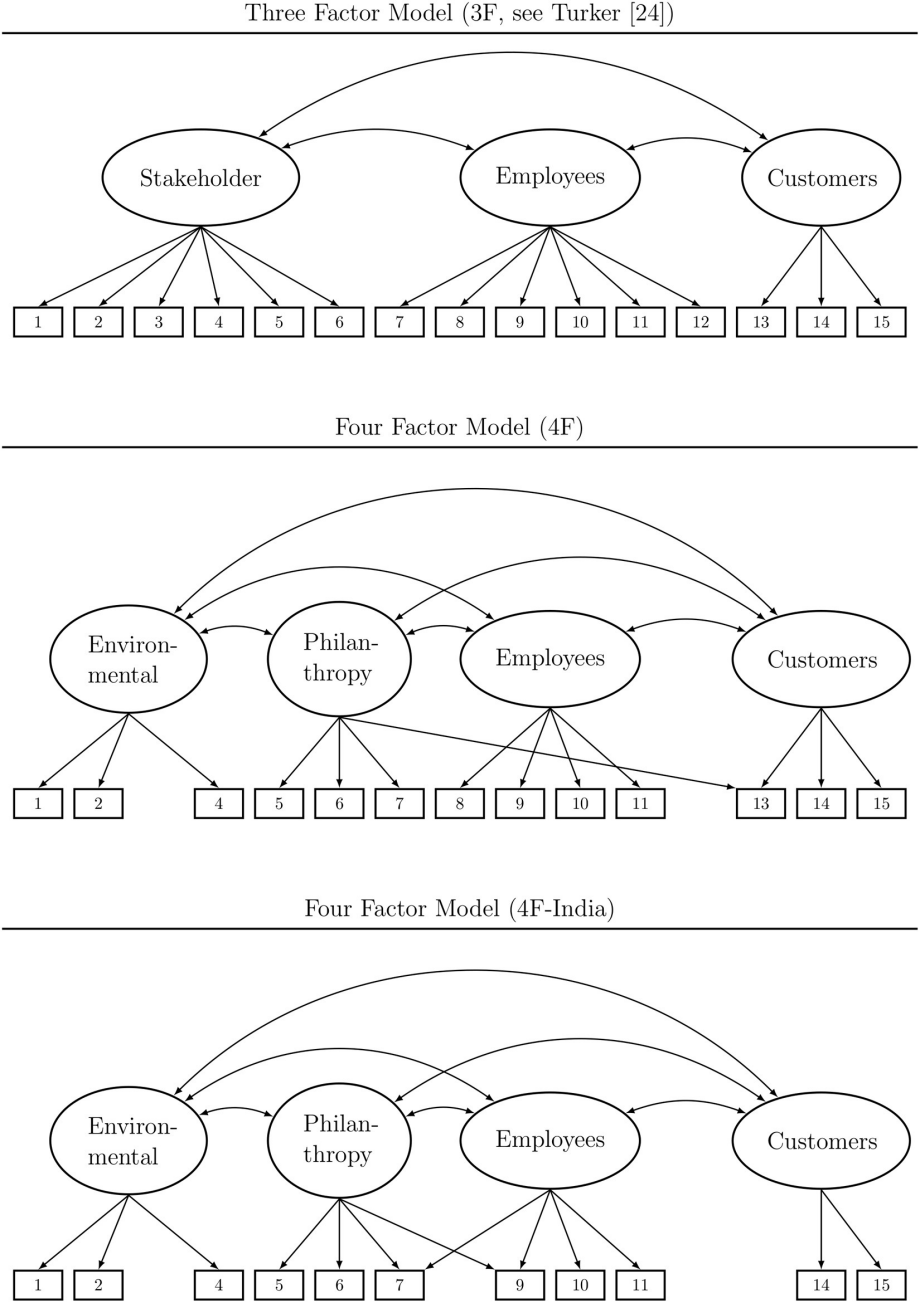
Factor models of the CSR scale. Items see Table 1.

The original three-factor structure (CSR to social and nonsocial stakeholders, CSR to employees and CSR to customers) of the CSR questionnaire by Turker [19] failed to match the data in all five samples. We tested the model as displayed in Fig 1, whereas we relaxed the assumption of independent factors as originally presumed by Turker [19]. With regard to the fit criteria by Hu and Bentler [31] as well as the results of the BSEMs, the original model displayed poor model fit (see Table 2). Further information on item means, variances, and loadings as well as covariance matrices can be found in the supporting information (S1 through S3 Tables).

**Table 2:** Model fits of the measurement models (CFA and BSEM).

CFA^a^										BSEM^b^					
Model	Sample	N	χ^2^	df	p	CFI	SRMR	RMSEA	90%-CI of RMSEA	# Para	95%-CrI of χ^2^	PPp	DIC	pD	T-σ^2^
3F	US-1	146	183.9	87	< .001	.884	.071	.087	.072–.102	78	65.03–156.16	< .001	6034.31	56.85	>1
	US-2	194	194.8	87	< .001	.916	.070	.080	.067–.092	78	95.08–171.8	< .001	7709.56	57.60	>1
	GER-1	155	243.2	87	< .001	.894	.087	.108	.093–.122	78	105.39–186.07	< .001	6464.14	59.65	>1
	GER-2	193	224.4	87	< .001	.883	.081	.090	.078–.103	78	82.49–161.48	< .001	8012.29	59.30	>1
	INDIA	432	440.35	87	< .001	.862	.082	.097	.089–.105	78	220.35–304.84	< .001	14996.49	65.13	>1
4F	US-1	146	82.9	58	.018	.963	.058	.054	.029–.076	84	-12.87–59.93	.098	5269.42	53.55	.008
	US-2	194	86.3	58	.009	.972	.054	.050	.030–.068	84	-7.62–61.53	.082	6676.11	58.03	.008
	GER-1	155	93.4	58	.002	.969	.046	.063	.040–.084	84	-6.76–57.98	.115	5662.21	54.28	.007
	GER-2	193	87.3	58	.008	.968	.045	.051	.030–.070	84	-6.14–69.49	.130	6999.27	55.86	.007
	INDIA	432	232.7	58	< .001	.910	.065	.083	.074–.093	84	35.73–112.31	< .001	12979.01	64.74	>1
4F-India	US-1	146	45.53	36	.133	.983	.037	.043	.000–.073	71	18.19–91.74	< .001	4523.03	40.95	.021
	US-2	194	33.90	36	.569	1.000	.033	.000	.000–.043	71	5.00–62.34	.009	5701.19	48.26	.011
	GER-1	155	47.35	36	.098	.987	.037	.045	.000–.074	71	-3.41–53.67	.045	4920.08	46.55	.008
	GER-2	193	38.29	36	.366	.997	.035	.018	.000–.052	71	10.76–67.92	.016	6076.70	43.75	.007
	INDIA	432	84.84	36	< .001	.970	.041	.056	.043–.069	71	15.07–98.73	< .001	11110.16	43.28	>1

Notes: BSEM-χ^2^ is the difference between observed and replicated χ^2^. CFI = Comparative fit index, SRMR = Standardized root mean residual, RMSEA = Root mean square error of approximation, # Para = Number of free parameters; CrI = Credibility interval; PPp = posterior predictive p value; DIC = deviance information criterion; pD = estimated number of parameters; T-σ^2^ = tipping-point cross-loading prior variance, resulting in PPp > .05.

^a^ CFA estimated with Satorra-Bentler-corrected χ^2^.

^b^ BSEMs tested with prior variances = .01.

#### Model 4F

We then searched for alternative measurement models with a better description of the factorial structure. Saris, Satorra, and Van der Veld [52] suggested the calculation of modification indices in an exploratory-confirmatory approach which led to the following procedure: Using the US-1 sample and maximum-likelihood-CFA techniques we investigated the modification indices of the item loadings as well as the residual covariances. The original model was altered stepwise starting with the modification promising the highest decrease in chi-square. Beside this statistical procedure, modifications had to be theoretically meaningful as well. When no further theoretically plausible alteration was possible, the measurement model was finally tested with the other samples to assure its validity and to avoid overfitting.

In this manner, we derived a model with a fourth factor (called philanthropy) and excluded items 3 and 12 in the process due to high residual correlations with other items indicating redundancy. As can be seen in Fig 1 the original three-factor structure by Turker [19] was altered only partially. The stakeholder factor was split into environmental and philanthropy aspects, whereas the employee factor kept its core items, and the customer factor remained the same aside from a single cross-loading. All item loadings and factor correlations were statistically significant, (see supplemental material). Following the results from the CFA and BSEM estimations, the 4F model displayed satisfying fit in all four Western samples, but not in the Indian sample (see Table 2). The two descendants of the stakeholder factor, environmental CSR and philanthropy, were rather strongly correlated in all samples (.77 < r < .88). In order to avoid an overfitted model, we compared model 4F with a simplified variant, where these two factors were melted into one and all other model properties were left unchanged. Likelihood ratio tests of for these two competing models revealed significant better fit of model 4F for all five samples (ps < .017). We thus concluded to keep four factors despite high factor correlations.

Moderate change of Turker’s [19] proposed measurement model thus led to a substantially improved description of the factor structure of the CSR questionnaire in four of the five samples.

#### Model 4F-India

In contrast to the Western samples, the modified factor model 4F did not fit the data in the Indian sample well (see Table 2). This divergence of the Indian sample from the factor structure in the other four samples motivated further exploratory modifications of the measurement model in order to find a common configural factor model. Based on the modification indices of the model 4F in the Indian sample, step-by-step alterations were undertaken following the procedure described above. To reach good fit indices in the CFA estimations, the exclusion of two more items (8 and 13) and alteration of the cross-loading of philanthropy was necessary as well as the inclusion of another cross-loading for the factor employee-related CSR. The final model 4F-India is displayed in Fig 1. In sum, major transformations had to be undertaken to Turker’s [19] original model to yield satisfactory fit indices in CFA for the Indian sample as well as the four other samples, which superseded those of model 4F (see Table 2). On the contrary, BSEM estimation did not provide evidence for a definite superiority of model 4F-India when compared to model 4F. In fact, in all samples the PPps of 4F-India were smaller and the threshold prior variances were higher, respectively. Both findings indicated a worse absolute fit. We want to point out, that BSEM provided no evidence for model fit even in the Indian sample, although we used this very sample for the presented modifications. The second step of model alteration thus yielded a stripped-down version of the CSR scale which found ambiguous evidence in the data.

### Measurement invariance

Here, only the four Western samples were considered as model 4F did not fit in the Indian sample and the altered model 4F-India displayed incongruous results. The basic presupposition of factorial validity was thus not met for the Indian sample.

The configural model showed sufficient model fit when estimated simultaneously across the four groups (see Table 3). When the loadings were constrained to equality, statistically insignificant change in chi-square was observed. Weak invariance was therefore accepted for the four Western groups.

**Table 3:** Measurement invariance across all five samples.

	invariance model	χ^2^	df	CFI	SRMR	RMSEA	KI RMSEA
4F	(1) configural	349.3*	232	.968	.051	.054	.044–.064
	(2) weak, 1 + loadings equal	384.8*	262	.966	.056	.052	.042–.062
	(Δ 2 − 1)	(33.7)	(30)	(-.002)	(.005)	(.002)	
	(3) strong, 2 + intercepts equal	561.8*	289	.925	.067	.074	.066–.082
	(Δ 3 − 2)	(341.6*)	(27)	(-.041)	(.011)	(.022)	
	(4) partial strong, ex. items 6, 9	443.0*	283	.956	.061	.057	.048–.066
	(Δ 4 − 2)	(72.1*)	(21)	(-.010)	(.005)	(.005)	
	(5) strong, US samples only	207.7*	135	.956	.062	.056	.043–.069
		(44.3*)	(9)	(-.012)	(.004)	(.007)	
	(6) strong, German samples only	208.3*	135	.964	.050	.056	.042–.069
		(24.2*)	(9)	(-.006)	(.002)	(.003)	
	(7) strict, 3 + residuals equal	639.0*	328	.915	.068	.074	.066–.082
	(Δ 7 − 3)	(77.1*)	(39)	(-.010)	(.001)	(.000)	

Notes: χ^2^ and Δ χ^2^ values are Satorra-Bentler adjusted. Positive numbers for Δ fit indices indicate increased values. CFI = Comparative fit index, SRMR = Standardized root mean residual, RMSEA = Root mean square error of approximation.

* p < .05.

In contrast, strong invariance could not be obtained (see Table 3). Restraining item intercepts to equality led to a statistically significant chi-square difference as well as a sizeable change in the fit indices. When examining the source of invariance regarding the intercepts, we found that items 6 and 9 were the most important causes, reducing noninvariance considerably with differences in the fit indices being close to their cut-offs. All other items differed only marginally, although affirmation was generally higher in the US samples, a trend which was reversed for items 6 and 9. We then hypothesized, that this could be due to shifting of meaning in the translated scale and that the two US and two German samples analyzed separately should thus display strong invariance. As can be seen in Table 3, this was the case. Although the change in the CFI exceeded the cut-off for the US-American samples, this decrease was not supplemented by another fit index showing noninvariance. Thus, the incapacity for strong invariance in the two above mentioned items was most likely caused by the translation from English to German.

As the stage of strong invariance could not be verified, strict invariance could not be obtained, too, although the change in fit indices was marginal and non-substantial when the item residuals were set equal. In summary, the factors contributed the same amount of variance to the items in the four Western samples, but some item intercepts varied across groups. Thus, only group comparisons of correlation coefficients are feasible, but comparisons of mean scores have no clear interpretation.

### Reliabilities

We calculated reliability estimates using model 4F because the alternative 4F-India was evaluated to be inferior. As weak measurement invariance was shown for the four Western samples, we calculated McDonald’s ω_h_ for the stacked data sets (n = 688) to get a precise and factorial valid reliability estimate. When a simple total score for all 13 items is computed by averaging, we calculated a mediocre to good reliability of ω_h_ = .77, whereas Cronbach’s α in comparison also accounts for variance not explained by the general factor and yielded α = .91. The four factors of model 4F displayed mostly good reliabilities considering the low number of items (environmental ω = .87, philanthropy ω = .78, employee ω = .84, customer ω = .66).

## Discussion

The present study examined the factorial validity of Turker’s [19] questionnaire for a self-assessment of perceived CSR.

### Strengths and limitations

When considering CSR, a relevant construct in a globalized economy and the employees’ perceptions of CSR an important aspect concerning acceptance and identification, multi-national evaluation samples are necessary as only equivalent and well-studied measures make cross-cultural comparisons feasible [47]. Originally Turker [19] suggested four factors, however we decided to omit the CSR to government items as they merely represent legally appropriate behavior [30]. With samples from three countries with economically different contexts we were able to study the factorial validity of the CSR scale presented by Turker [19] in depth. Importantly, the samples were comparable regarding their basic demographic properties like gender, age, education, and industry branch. The five samples allowed for tests along two important lines of measurement invariance: language and socio-economic background. Differences of the English and German version could be investigated (comparing all English-speaking samples from the US and India to the German speaking samples) as well as differences between socio-economic contexts (Western samples compared to the Indian sample).

Drawing from earlier work by Turker [19], Newman et al. [23], and Dange et al. [30], we carried out the analyses in a confirmatory manner and tested specific factors structures. When the originally assumed model failed, the multiple samples allowed for modifications, which could then be put to the test as well. Beyond traditional maximum likelihood factor analysis, BSEM was used as a mathematically more advanced approach to overcome theoretical limitations of standard CFA. Most importantly in comparison, CFA assumes completely independent clusters of items in multi-dimensional scales. As this prerequisite has been criticized to be unsuitable to some psychological constructs [39], techniques accounting for more heterogeneous constructs are in need. Strict independence is replaced in BSEM by stochastic independence of the item clusters. On the downside some authors argue that BSEM might blur strong theoretical foundations like independent item clusters as these assumptions simply reflect the call for well designed indicators [53]. Taking their perspective a revision of the full questionnaire would be in need regarding the incapacity of the original CSR factor model. Although this represents a sound option, we wanted to gather further information on the measurement properties of the current items first. Thus, the application of two different methods proved useful for the evaluation of the factor structure, especially in case of the Indian sample.

On the other hand, as the results are based on only three countries, the present study is merely a first step in evaluating measurement invariance and additional research is needed incorporating data from other regions like Africa and South America. Further methodological limitations arise from the sampling procedures. The subjects were recruited online in a non-probabilistic way through online networks and Amazon mechanical Turk. Although severe doubts about inferences drawn from internet-based samples are most likely incongruous [27,54], other sampling designs like surveying subjects from a few enterprises of the same industrial branch in different countries may be superior.

Concerning the different language versions of the questionnaire, the use of back-translation techniques has been criticized [55]. It seems that item 6 does not fit in the German and the American culture equally. It would therefore be useful in the future to follow guidelines as introduced e.g. in Hambleton [56].

### Findings

Only an altered version (4F) containing four factors (CSR to customers, CSR to employees, environmental CSR, and philanthropy, for origin of terms see [57]) and two excluded items reached acceptable fit maximum-likelihood CFA and BSEM. This did not account for the Indian sample in contrast to the four Western samples from the US and Germany. Only a second step of model modification with the exclusion of two more items (4F-India) led to appropriate CFA fit indices in the Indian sample, but it constitutes a theoretically and in sum empirically weaker model when the poor BSEM results are incorporated. Thus, Model 4F is to be preferred, as its modifications are theoretically justified and its validity can be shown for the US American and German samples.

The measurement invariance tests concerned the equivalence of the CSR scale with respect to different language versions and different socio-economic backgrounds. In detail, weak invariance was found for the Western samples with model 4F. This stage of invariance allows for comparisons of regression slopes or correlations across groups or studies. To compare mean scores between different groups, strong or strict invariance would be necessary. These higher levels could not be demonstrated without allowing some item intercepts to vary freely. I.e., differences in latent mean scores between US-american and German employees could not be meaningfully interpreted. Analyzing the groups separated by language and socio-economic background marked both factors to be the most likely reasons for the lack of strong invariance. Differences between the English and the German version in the item "My organization contributes to campaigns and projects that promote the well-being of society" may be due to a shift of meaning, as the German wording mentions the development rather than the well-being of society. The same accounts for "The management of my organization is primarily concerned with employees’ needs and wants." as the German item left out the emphasis of "primarily". Still, these considerations do not generalize to the Indian sample, which answered the same English version. Item difficulties varied in the three English samples, while the German samples were homogeneous. Incorporating the findings of varying item residuals in the five samples, but invariant item residuals in the four Western samples, tests for mean differences would thus only be adequate as long as Western samples are examined and minor differences of two items are taken into account taken into account or different item translations are put to the test. The current German version represents the first examined German translation of the CSR scale and displayed good, although not perfect item properties.

In summary, the concept of CSR was probably perceived differently in the Indian sample. Another explanation for the model misfit could be a different use of the English language in India. Our findings would thus not necessarily point to differences on conceptual level but rather indicate linguistic peculiarities. Translations to Hindi (or other Indian languages) would allow examining this matter.

Hence, at this moment, we can only draw conclusions on the factorial structure of this CSR measure for Western cultures. This would support Turker’s [19] assumption that the Turkish business communities converge to the European context. Although she underlined that Turkey has a unique position between Eastern and Western cultures and hence represents both, our results suggest that the construct of CSR as measured here rather refers to Western contexts. However, Dange et al. [30] adapted Turker’s questionnaire to the Indian culture. They also omitted the CSR to government items and replaced them with voluntary CSR to government behaviors. Furthermore, they added items to include CSR to shareholders. Exploratory factor analyses supported their expected structure and provided six factors that represent different stakeholders. Hence their adaption to the Indian context worked well, although they did not display a rigorous confirmatory analysis and used slightly different item wordings.

Turker’s CSR questionnaire has also been adapted for the Chinese context [22,23]. The exploration of the underlying factor structure yielded mixed results. Newman et al. [23] found support for the four-factor structure of CSR in their CFA on all study variables, whereas the exploratory factor analysis performed by Hofman et al. [22] suggested three factors named CSR to society, CSR to employees and CSR to government and customers. We therefore conclude that, all in all, in order to use Turker’s [19] CSR questionnaire in India, China or more generally Asian countries, a scale validation for these cultural contexts is still in need.

Turker’s [19] questionnaire is a useful measure to assess perceived CSR for research on the individual level. The proposed modifications could help organizational researchers getting insight into individual effects of CSR. On the one hand, this aim is hindered by the necessity of several versions in multiple languages to cover the global and cross-cultural aspect of CSR research. Translation and scale evaluation are thus important issues to allow sound conclusions. On the other hand, socio-economic diversity itself may change the perception and relevance of CSR in specific countries. Hence, a full picture is only possible when these characteristics are understood before relating CSR to other constructs or comparing groups. In CSR research, Turker’s [19] scale represents a step towards measuring individually perceived CSR activities, but it needs further development to satisfy the requirements of cross-cultural research. If invariance of a single scale cannot be obtained in the future, culture-specific questionnaires might pose a remedy.

## Supporting information

S1 Table Descriptive statistics: Item means and standard deviations. (DOCX)

S2 Table CFA, model 4F: Standardized item loadings and factor correlations (with standard errors). All reported parameters are significant with *p* < .01.
(DOCX)

S3 Table Covariance matrices of all items in all samples. (DOCX)
