# Supplementary material for: Comparative organizational research starts with sound measurement: Validity and invariance of Turker’s corporate social responsibility scale in five cross-cultural samples
Source: PLoS One. 2018 Nov 19;13(11):e0207331. doi: 10.1371/journal.pone.0207331 (PMC6242312; doi:10.1371/journal.pone.0207331)
Supplement: S1 Table — (DOCX) [file pone.0207331.s001.docx]

**Table S1. Descriptive statistics: Item means and standard deviations.**

| Item # | US-1 | US-2 | GER-1 | GER-2 | INDIA |
| --- | --- | --- | --- | --- | --- |
| # 1 | 3.26 (1.27) | 3.48 (1.23) | 2.84 (1.37) | 2.83 (1.32) | 3.31 (1.08) |
| # 2 | 3.51 (1.27) | 3.39 (1.27) | 2.82 (1.37) | 3.07 (1.36) | 3.19 (1.08) |
| # 3 | 3.32 (1.31) | 3.25 (1.27) | 2.95 (1.40) | 2.95 (1.29) | 3.25 (1.03) |
| # 4 | 3.15 (1.19) | 3.16 (1.21) | 2.72 (1.32) | 2.93 (1.28) | 3.24 (1.07) |
| # 5 | 3.10 (1.26) | 3.34 (1.18) | 2.37 (1.37) | 2.28 (1.20) | 2.84 (1.01) |
| # 6 | 3.47 (1.25) | 3.55 (1.23) | 3.13 (1.48) | 3.27 (1.31) | 3.22 (1.01) |
| # 7 | 3.73 (1.28) | 3.55 (1.25) | 2.70 (1.41) | 2.74 (1.36) | 3.50 (0.97) |
| # 8 | 3.77 (1.18) | 3.69 (1.10) | 3.42 (1.29) | 3.39 (1.11) | 3.88 (0.91) |
| # 9 | 3.17 (1.31) | 2.97 (1.20) | 3.23 (1.30) | 3.15 (1.11) | 3.23 (1.13) |
| # 10 | 3.49 (1.25) | 3.58 (1.15) | 3.25 (1.41) | 3.20 (1.28) | 3.57 (0.97) |
| # 11 | 3.64 (1.21) | 3.75 (1.08) | 3.21 (1.17) | 3.43 (1.03) | 3.67 (0.86) |
| # 12 | 3.90 (1.09) | 3.75 (1.21) | 3.57 (1.17) | 3.42 (1.12) | 3.36 (1.03) |
| # 13 | 3.35 (1.21) | 3.27 (1.12) | 2.81 (1.15) | 2.94 (1.02) | 3.40 (0.90) |
| # 14 | 3.88 (1.06) | 4.02 (0.94) | 3.55 (1.19) | 3.72 (1.13) | 3.95 (0.71) |
| # 15 | 4.18 (1.09) | 4.32 (0.82) | 4.08 (1.12) | 4.12 (1.05) | 4.20 (0.68) |
