# Supplementary material for: Comparative organizational research starts with sound measurement: Validity and invariance of Turker’s corporate social responsibility scale in five cross-cultural samples
Source: PLoS One. 2018 Nov 19;13(11):e0207331. doi: 10.1371/journal.pone.0207331 (PMC6242312; doi:10.1371/journal.pone.0207331)
Supplement: S2 Table — All reported parameters are significant with p < .01. (DOCX) [file pone.0207331.s002.docx]

**Table S2. CFA, model 4F: Standardized item loadings and factor correlations (with standard errors).** All reported parameters are significant with *p* < .01.

|  |  | US-1 | US-2 | GER-1 | GER-2 | INDIA |
| --- | --- | --- | --- | --- | --- | --- |
| **Item loadings** |  |  |  |  |  |  |
| Factor | Item # | λ | | | | |
| Environmental | # 1 | .70 | .82 | .84 | .79 | .80 |
|  | # 2 | .83 | .82 | .88 | .87 | .83 |
|  | # 4 | .84 | .77 | .87 | .85 | .90 |
| Philantrophy | # 5 | .75 | .71 | .69 | .62 | .61 |
|  | # 6 | .80 | .84 | .77 | .78 | .74 |
|  | # 7 | .67 | .64 | .78 | .68 | .66 |
|  | # 13 | .33 | .47 | .50 | .48 | .47 |
| Employees | # 8 | .62 | .68 | .83 | .67 | .72 |
|  | # 9 | .74 | .73 | .92 | .82 | .77 |
|  | # 11 | .85 | .80 | .80 | .73 | .55 |
|  | # 10 | .85 | .85 | .64 | .68 | .67 |
| Customers | # 13 | .53 | .22 | .37 | .38 | .22 |
|  | # 14 | .82 | .76 | .90 | .84 | .74 |
|  | # 15 | .69 | .78 | .69 | .65 | .77 |
| **Factor correlations** | |  |  |  |  |  |
| Factor 1 | Factor 2 | *r* | | | | |
| Environmental | Philantrophy | .88 | .81 | .88 | .78 | .77 |
| Environmental | Employees | .59 | .64 | .53 | .56 | .56 |
| Environmental | Customers | .48 | .49 | .50 | .48 | .43 |
| Philantrophy | Employees | .63 | .61 | .63 | .65 | .74 |
| Philantrophy | Customers | .57 | .51 | .46 | .29 | .43 |
| Employees | Customers | .67 | .68 | .57 | .50 | .63 |
