# Supplementary material for: Comparative organizational research starts with sound measurement: Validity and invariance of Turker’s corporate social responsibility scale in five cross-cultural samples
Source: PLoS One. 2018 Nov 19;13(11):e0207331. doi: 10.1371/journal.pone.0207331 (PMC6242312; doi:10.1371/journal.pone.0207331)
Supplement: S3 Table — (DOCX) [file pone.0207331.s003.docx]

**Table S3. Covariance matrices of all items in all samples.**

| **US-1** |  |  |  |  |  |  |  |  |  |  |  |  |  |  |  |
| --- | --- | --- | --- | --- | --- | --- | --- | --- | --- | --- | --- | --- | --- | --- | --- |
| Item # | 1 | 2 | 3 | 4 | 5 | 6 | 7 | 8 | 9 | 10 | 11 | 12 | 13 | 14 | 15 |
| 1 | 1.60 | 0.94 | 1.36 | 0.84 | 0.80 | 0.84 | 0.74 | 0.54 | 0.45 | 0.49 | 0.43 | 0.52 | 0.48 | 0.29 | 0.39 |
| 2 | 0.94 | 1.62 | 0.99 | 1.07 | 0.81 | 0.91 | 0.77 | 0.70 | 0.64 | 0.63 | 0.67 | 0.64 | 0.55 | 0.42 | 0.30 |
| 3 | 1.36 | 0.99 | 1.71 | 0.91 | 0.87 | 0.87 | 0.72 | 0.59 | 0.50 | 0.64 | 0.55 | 0.53 | 0.56 | 0.35 | 0.39 |
| 4 | 0.84 | 1.07 | 0.91 | 1.43 | 0.89 | 0.89 | 0.70 | 0.59 | 0.60 | 0.60 | 0.57 | 0.58 | 0.73 | 0.49 | 0.46 |
| 5 | 0.80 | 0.81 | 0.87 | 0.89 | 1.59 | 0.95 | 0.78 | 0.60 | 0.60 | 0.55 | 0.53 | 0.55 | 0.77 | 0.38 | 0.29 |
| 6 | 0.84 | 0.91 | 0.87 | 0.89 | 0.95 | 1.56 | 0.85 | 0.69 | 0.43 | 0.60 | 0.60 | 0.60 | 0.73 | 0.42 | 0.48 |
| 7 | 0.74 | 0.77 | 0.72 | 0.70 | 0.78 | 0.85 | 1.65 | 0.81 | 0.36 | 0.68 | 0.71 | 0.68 | 0.70 | 0.63 | 0.50 |
| 8 | 0.54 | 0.70 | 0.59 | 0.59 | 0.60 | 0.69 | 0.81 | 1.40 | 0.69 | 0.68 | 0.77 | 0.79 | 0.60 | 0.53 | 0.55 |
| 9 | 0.45 | 0.64 | 0.50 | 0.60 | 0.60 | 0.43 | 0.36 | 0.69 | 1.73 | 1.10 | 0.99 | 0.51 | 0.63 | 0.45 | 0.44 |
| 10 | 0.49 | 0.63 | 0.64 | 0.60 | 0.55 | 0.60 | 0.68 | 0.68 | 1.10 | 1.56 | 1.10 | 0.55 | 0.74 | 0.57 | 0.58 |
| 11 | 0.43 | 0.67 | 0.55 | 0.57 | 0.53 | 0.60 | 0.71 | 0.77 | 0.99 | 1.10 | 1.47 | 0.61 | 0.71 | 0.61 | 0.53 |
| 12 | 0.52 | 0.64 | 0.53 | 0.58 | 0.55 | 0.60 | 0.68 | 0.79 | 0.51 | 0.55 | 0.61 | 1.19 | 0.61 | 0.56 | 0.56 |
| 13 | 0.48 | 0.55 | 0.56 | 0.73 | 0.77 | 0.73 | 0.70 | 0.60 | 0.63 | 0.74 | 0.71 | 0.61 | 1.46 | 0.77 | 0.60 |
| 14 | 0.29 | 0.42 | 0.35 | 0.49 | 0.38 | 0.42 | 0.63 | 0.53 | 0.45 | 0.57 | 0.61 | 0.56 | 0.77 | 1.12 | 0.66 |
| 15 | 0.39 | 0.30 | 0.39 | 0.46 | 0.29 | 0.48 | 0.50 | 0.55 | 0.44 | 0.58 | 0.53 | 0.56 | 0.60 | 0.66 | 1.18 |

| **US-2** |  |  |  |  |  |  |  |  |  |  |  |  |  |  |  |
| --- | --- | --- | --- | --- | --- | --- | --- | --- | --- | --- | --- | --- | --- | --- | --- |
| Item # | 1 | 2 | 3 | 4 | 5 | 6 | 7 | 8 | 9 | 10 | 11 | 12 | 13 | 14 | 15 |
| 1 | 1.50 | 1.05 | 1.10 | 0.94 | 0.64 | 0.83 | 0.70 | 0.39 | 0.68 | 0.58 | 0.50 | 0.62 | 0.62 | 0.35 | 0.31 |
| 2 | 1.05 | 1.61 | 1.07 | 0.97 | 0.65 | 0.85 | 0.76 | 0.59 | 0.69 | 0.53 | 0.53 | 0.84 | 0.70 | 0.34 | 0.30 |
| 3 | 1.10 | 1.07 | 1.62 | 1.05 | 0.79 | 0.95 | 0.71 | 0.54 | 0.62 | 0.64 | 0.47 | 0.58 | 0.56 | 0.45 | 0.26 |
| 4 | 0.94 | 0.97 | 1.05 | 1.48 | 0.63 | 0.70 | 0.62 | 0.60 | 0.71 | 0.56 | 0.49 | 0.50 | 0.61 | 0.39 | 0.26 |
| 5 | 0.64 | 0.65 | 0.79 | 0.63 | 1.40 | 0.96 | 0.57 | 0.41 | 0.48 | 0.40 | 0.33 | 0.45 | 0.51 | 0.26 | 0.22 |
| 6 | 0.83 | 0.85 | 0.95 | 0.70 | 0.96 | 1.51 | 0.82 | 0.51 | 0.54 | 0.57 | 0.45 | 0.72 | 0.60 | 0.42 | 0.32 |
| 7 | 0.70 | 0.76 | 0.71 | 0.62 | 0.57 | 0.82 | 1.55 | 0.55 | 0.49 | 0.53 | 0.47 | 0.67 | 0.55 | 0.42 | 0.35 |
| 8 | 0.39 | 0.59 | 0.54 | 0.60 | 0.41 | 0.51 | 0.55 | 1.22 | 0.64 | 0.74 | 0.60 | 0.78 | 0.61 | 0.45 | 0.28 |
| 9 | 0.68 | 0.69 | 0.62 | 0.71 | 0.48 | 0.54 | 0.49 | 0.64 | 1.44 | 0.84 | 0.78 | 0.66 | 0.73 | 0.31 | 0.28 |
| 10 | 0.58 | 0.53 | 0.64 | 0.56 | 0.40 | 0.57 | 0.53 | 0.74 | 0.84 | 1.31 | 0.85 | 0.73 | 0.59 | 0.47 | 0.45 |
| 11 | 0.50 | 0.53 | 0.47 | 0.49 | 0.33 | 0.45 | 0.47 | 0.60 | 0.78 | 0.85 | 1.17 | 0.61 | 0.51 | 0.41 | 0.41 |
| 12 | 0.62 | 0.84 | 0.58 | 0.50 | 0.45 | 0.72 | 0.67 | 0.78 | 0.66 | 0.73 | 0.61 | 1.47 | 0.58 | 0.48 | 0.34 |
| 13 | 0.62 | 0.70 | 0.56 | 0.61 | 0.51 | 0.60 | 0.55 | 0.61 | 0.73 | 0.59 | 0.51 | 0.58 | 1.26 | 0.32 | 0.31 |
| 14 | 0.35 | 0.34 | 0.45 | 0.39 | 0.26 | 0.42 | 0.42 | 0.45 | 0.31 | 0.47 | 0.41 | 0.48 | 0.32 | 0.89 | 0.47 |
| 15 | 0.31 | 0.30 | 0.26 | 0.26 | 0.22 | 0.32 | 0.35 | 0.28 | 0.28 | 0.45 | 0.41 | 0.34 | 0.31 | 0.47 | 0.67 |

| **GER-1** |  |  |  |  |  |  |  |  |  |  |  |  |  |  |  |
| --- | --- | --- | --- | --- | --- | --- | --- | --- | --- | --- | --- | --- | --- | --- | --- |
| Item # | 1 | 2 | 3 | 4 | 5 | 6 | 7 | 8 | 9 | 10 | 11 | 12 | 13 | 14 | 15 |
| 1 | 1.88 | 1.38 | 1.60 | 1.31 | 1.00 | 1.09 | 1.06 | 0.72 | 0.52 | 0.58 | 0.52 | 0.57 | 0.85 | 0.64 | 0.40 |
| 2 | 1.38 | 1.86 | 1.22 | 1.38 | 0.97 | 1.22 | 1.17 | 0.88 | 0.77 | 0.70 | 0.66 | 0.69 | 0.85 | 0.59 | 0.26 |
| 3 | 1.60 | 1.22 | 1.95 | 1.39 | 0.91 | 1.09 | 0.98 | 0.69 | 0.60 | 0.65 | 0.58 | 0.63 | 0.97 | 0.69 | 0.58 |
| 4 | 1.31 | 1.38 | 1.39 | 1.75 | 0.93 | 1.04 | 1.06 | 0.67 | 0.66 | 0.64 | 0.61 | 0.61 | 1.00 | 0.69 | 0.47 |
| 5 | 1.00 | 0.97 | 0.91 | 0.93 | 1.87 | 1.24 | 0.91 | 0.63 | 0.58 | 0.78 | 0.65 | 0.47 | 0.72 | 0.53 | 0.35 |
| 6 | 1.09 | 1.22 | 1.09 | 1.04 | 1.24 | 2.18 | 1.27 | 0.84 | 0.69 | 0.81 | 0.56 | 0.72 | 0.81 | 0.53 | 0.47 |
| 7 | 1.06 | 1.17 | 0.98 | 1.06 | 0.91 | 1.27 | 1.98 | 0.94 | 0.94 | 0.86 | 0.69 | 0.81 | 0.82 | 0.49 | 0.40 |
| 8 | 0.72 | 0.88 | 0.69 | 0.67 | 0.63 | 0.84 | 0.94 | 1.67 | 1.31 | 0.89 | 0.92 | 1.04 | 0.59 | 0.65 | 0.52 |
| 9 | 0.52 | 0.77 | 0.60 | 0.66 | 0.58 | 0.69 | 0.94 | 1.31 | 1.70 | 1.03 | 1.14 | 0.96 | 0.60 | 0.67 | 0.52 |
| 10 | 0.58 | 0.70 | 0.65 | 0.64 | 0.78 | 0.81 | 0.86 | 0.89 | 1.03 | 1.98 | 0.95 | 0.96 | 0.62 | 0.62 | 0.46 |
| 11 | 0.52 | 0.66 | 0.58 | 0.61 | 0.65 | 0.56 | 0.69 | 0.92 | 1.14 | 0.95 | 1.38 | 0.75 | 0.50 | 0.66 | 0.53 |
| 12 | 0.57 | 0.69 | 0.63 | 0.61 | 0.47 | 0.72 | 0.81 | 1.04 | 0.96 | 0.96 | 0.75 | 1.36 | 0.51 | 0.60 | 0.56 |
| 13 | 0.85 | 0.85 | 0.97 | 1.00 | 0.72 | 0.81 | 0.82 | 0.59 | 0.60 | 0.62 | 0.50 | 0.51 | 1.31 | 0.72 | 0.56 |
| 14 | 0.64 | 0.59 | 0.69 | 0.69 | 0.53 | 0.53 | 0.49 | 0.65 | 0.67 | 0.62 | 0.66 | 0.60 | 0.72 | 1.41 | 0.82 |
| 15 | 0.40 | 0.26 | 0.58 | 0.47 | 0.35 | 0.47 | 0.40 | 0.52 | 0.52 | 0.46 | 0.53 | 0.56 | 0.56 | 0.82 | 1.25 |

| **GER-2** |  |  |  |  |  |  |  |  |  |  |  |  |  |  |  |
| --- | --- | --- | --- | --- | --- | --- | --- | --- | --- | --- | --- | --- | --- | --- | --- |
| Item # | 1 | 2 | 3 | 4 | 5 | 6 | 7 | 8 | 9 | 10 | 11 | 12 | 13 | 14 | 15 |
| 1 | 1.75 | 1.27 | 1.34 | 1.14 | 0.56 | 0.78 | 0.68 | 0.48 | 0.37 | 0.61 | 0.43 | 0.52 | 0.60 | 0.50 | 0.25 |
| 2 | 1.27 | 1.85 | 1.17 | 1.27 | 0.60 | 1.08 | 0.86 | 0.64 | 0.53 | 0.69 | 0.46 | 0.56 | 0.59 | 0.51 | 0.26 |
| 3 | 1.34 | 1.17 | 1.67 | 1.21 | 0.59 | 0.72 | 0.59 | 0.53 | 0.34 | 0.56 | 0.40 | 0.51 | 0.63 | 0.53 | 0.34 |
| 4 | 1.14 | 1.27 | 1.21 | 1.64 | 0.61 | 0.88 | 0.69 | 0.58 | 0.48 | 0.66 | 0.43 | 0.59 | 0.79 | 0.59 | 0.30 |
| 5 | 0.56 | 0.60 | 0.59 | 0.61 | 1.45 | 0.76 | 0.74 | 0.41 | 0.49 | 0.43 | 0.35 | 0.36 | 0.43 | 0.19 | 0.06 |
| 6 | 0.78 | 1.08 | 0.72 | 0.88 | 0.76 | 1.73 | 0.98 | 0.61 | 0.57 | 0.70 | 0.38 | 0.55 | 0.58 | 0.39 | 0.15 |
| 7 | 0.68 | 0.86 | 0.59 | 0.69 | 0.74 | 0.98 | 1.84 | 0.57 | 0.47 | 0.45 | 0.35 | 0.62 | 0.57 | 0.22 | 0.15 |
| 8 | 0.48 | 0.64 | 0.53 | 0.58 | 0.41 | 0.61 | 0.57 | 1.24 | 0.68 | 0.58 | 0.56 | 0.70 | 0.46 | 0.35 | 0.10 |
| 9 | 0.37 | 0.53 | 0.34 | 0.48 | 0.49 | 0.57 | 0.47 | 0.68 | 1.24 | 0.81 | 0.72 | 0.57 | 0.41 | 0.37 | 0.37 |
| 10 | 0.61 | 0.69 | 0.56 | 0.66 | 0.43 | 0.70 | 0.45 | 0.58 | 0.81 | 1.65 | 0.62 | 0.68 | 0.57 | 0.49 | 0.34 |
| 11 | 0.43 | 0.46 | 0.40 | 0.43 | 0.35 | 0.38 | 0.35 | 0.56 | 0.72 | 0.62 | 1.06 | 0.58 | 0.44 | 0.37 | 0.27 |
| 12 | 0.52 | 0.56 | 0.51 | 0.59 | 0.36 | 0.55 | 0.62 | 0.70 | 0.57 | 0.68 | 0.58 | 1.26 | 0.47 | 0.31 | 0.23 |
| 13 | 0.60 | 0.59 | 0.63 | 0.79 | 0.43 | 0.58 | 0.57 | 0.46 | 0.41 | 0.57 | 0.44 | 0.47 | 1.04 | 0.50 | 0.34 |
| 14 | 0.50 | 0.51 | 0.53 | 0.59 | 0.19 | 0.39 | 0.22 | 0.35 | 0.37 | 0.49 | 0.37 | 0.31 | 0.50 | 1.28 | 0.66 |
| 15 | 0.25 | 0.26 | 0.34 | 0.30 | 0.06 | 0.15 | 0.15 | 0.10 | 0.37 | 0.34 | 0.27 | 0.23 | 0.34 | 0.66 | 1.10 |

| **INDIA** |  |  |  |  |  |  |  |  |  |  |  |  |  |  |  |
| --- | --- | --- | --- | --- | --- | --- | --- | --- | --- | --- | --- | --- | --- | --- | --- |
| Item # | 1 | 2 | 3 | 4 | 5 | 6 | 7 | 8 | 9 | 10 | 11 | 12 | 13 | 14 | 15 |
| 1 | 1.17 | 0.76 | 0.73 | 0.82 | 0.50 | 0.57 | 0.44 | 0.36 | 0.54 | 0.28 | 0.06 | 0.62 | 0.38 | 0.19 | 0.22 |
| 2 | 0.76 | 1.17 | 0.63 | 0.87 | 0.48 | 0.52 | 0.31 | 0.30 | 0.48 | 0.27 | 0.09 | 0.58 | 0.37 | 0.19 | 0.23 |
| 3 | 0.73 | 0.63 | 1.06 | 0.63 | 0.41 | 0.51 | 0.33 | 0.26 | 0.53 | 0.34 | 0.05 | 0.49 | 0.35 | 0.11 | 0.15 |
| 4 | 0.82 | 0.87 | 0.63 | 1.14 | 0.48 | 0.52 | 0.41 | 0.35 | 0.56 | 0.34 | 0.15 | 0.65 | 0.43 | 0.18 | 0.22 |
| 5 | 0.50 | 0.48 | 0.41 | 0.48 | 1.03 | 0.54 | 0.33 | 0.27 | 0.33 | 0.18 | 0.07 | 0.50 | 0.34 | 0.09 | 0.14 |
| 6 | 0.57 | 0.52 | 0.51 | 0.52 | 0.54 | 1.03 | 0.49 | 0.38 | 0.51 | 0.26 | 0.07 | 0.54 | 0.34 | 0.15 | 0.17 |
| 7 | 0.44 | 0.31 | 0.33 | 0.41 | 0.33 | 0.49 | 0.94 | 0.52 | 0.48 | 0.40 | 0.28 | 0.44 | 0.31 | 0.20 | 0.17 |
| 8 | 0.36 | 0.30 | 0.26 | 0.35 | 0.27 | 0.38 | 0.52 | 0.82 | 0.54 | 0.36 | 0.31 | 0.46 | 0.29 | 0.25 | 0.25 |
| 9 | 0.54 | 0.48 | 0.53 | 0.56 | 0.33 | 0.51 | 0.48 | 0.54 | 1.28 | 0.61 | 0.37 | 0.70 | 0.48 | 0.25 | 0.27 |
| 10 | 0.28 | 0.27 | 0.34 | 0.34 | 0.18 | 0.26 | 0.40 | 0.36 | 0.61 | 0.94 | 0.46 | 0.40 | 0.25 | 0.21 | 0.16 |
| 11 | 0.06 | 0.09 | 0.05 | 0.15 | 0.07 | 0.07 | 0.28 | 0.31 | 0.37 | 0.46 | 0.74 | 0.23 | 0.16 | 0.23 | 0.17 |
| 12 | 0.62 | 0.58 | 0.49 | 0.65 | 0.50 | 0.54 | 0.44 | 0.46 | 0.70 | 0.40 | 0.23 | 1.07 | 0.52 | 0.19 | 0.22 |
| 13 | 0.38 | 0.37 | 0.35 | 0.43 | 0.34 | 0.34 | 0.31 | 0.29 | 0.48 | 0.25 | 0.16 | 0.52 | 0.82 | 0.19 | 0.20 |
| 14 | 0.19 | 0.19 | 0.11 | 0.18 | 0.09 | 0.15 | 0.20 | 0.25 | 0.25 | 0.21 | 0.23 | 0.19 | 0.19 | 0.51 | 0.28 |
| 15 | 0.22 | 0.23 | 0.15 | 0.22 | 0.14 | 0.17 | 0.17 | 0.25 | 0.27 | 0.16 | 0.17 | 0.22 | 0.20 | 0.28 | 0.46 |
